# A 0.002-mm^2^, 2.9-μW Pulse-Frequency-Modulation-Based Temperature Sensor for Wide Sensing Range from −60 °C to 120 °C †

**DOI:** 10.3390/s26175506

**Published:** 2026-08-30

**Authors:** Chiyuan Zhang, Nan Chen, Fang Zhu, Shanshan Li, Libin Yao, Yuxuan Luo

**Affiliations:** 1 Kunming Institute of Physics, Kunming 650223, China; z656866760@163.com (C.Z.); zhufangnn@163.com (F.Z.); lss_angela@163.com (S.L.); 15825298916@163.com (L.Y.); 2 College of Integrated Circuits, Zhejiang University, Hangzhou 310027, China; luoyx@zju.edu.cn

**Keywords:** CMOS temperature sensor, PFM, wide temperature range, small area, low power

## Abstract

**Highlights:**

**What are the main findings?**

- A fully CMOS PFM-based temperature sensor is developed, featuring a wide sensing range from −60 °C to 120 °C, 2.9 μW power consumption, and 2200 μm^2^ area in 180 nm CMOS.
- Improved cascode current mirror and monostable circuit enhance linearity, stabilize oscillation, and extend the operating temperature range.

**What are the implications of the main findings?**

- The quasi-digital PFM architecture enables excellent scalability to advanced processes for ultra-low-power and compact thermal sensing.
- The wide-range, high energy-efficiency features facilitate aerospace, industrial, and cryogenic infrared detector applications.

**Abstract:**

This work presents a compact, low-power fully CMOS pulse-frequency-modulation (PFM) temperature sensor fabricated in 180 nm technology, supporting a wide −60 °C to 120 °C sensing range. Subthreshold biasing generates temperature-proportional current, while PFM converts current variations into digital pulse signals to bypass supply voltage swing limitations. The following two core optimizations are proposed: an improved cascode current mirror to suppress channel-length modulation and stabilize thermal current, and a temperature-insensitive monostable circuit eliminating extreme-temperature oscillation failure caused by variable reset pulse width. A four-point off-chip calibration model further compensates static and temperature-dependent loop delay errors. The prototype occupies only 2200 μm^2^ core area and consumes 2.9 μW. With a 5 ms conversion time, it achieves 47 mK RMS temperature resolution, 32 pJ·K^2^ resolution FoM and 0.06 pJ·K^−2^·mm^−2^ area FoM, with calibrated measurement inaccuracy limited to +1.8/−1.9 °C (3σ). This RC-free quasi-digital PFM architecture scales well to advanced process nodes and fits aerospace, cryogenic infrared detector and low-power industrial thermal sensing applications.

## 1. Introduction

Precise temperature measurement and regulation facilitate optimal operation of equipment and instruments and ensure the smooth progress of experiments and chemical reactions. Temperature sensors supporting stable temperature control are widely deployed across numerous fields, including biomedical devices, household appliances, and even aerospace equipment. Most temperature sensors perform reliably under standard conditions. However, they still face constraints in specialized applications (e.g., aerospace systems, bio/chemical labs) with strict limits on measurement range (−50 °C to 120 °C for industrial variants), miniaturization, and power efficiency. Notably, these sensors play a vital role in calibration and thermal stabilization for cryogenic detectors operating in the −60 °C to −40 °C range, such as those used in infrared imaging or quantum computing systems. Maintaining precise temperature feedback prevents detector performance degradation arising from thermal drift, while safeguarding critical components from damage under ultra-low-temperature conditions. These environments often expose sensors to extreme thermal fluctuations, demanding robust adaptability, especially under circumstances involving rapid temperature transitions from cryogenic levels to ambient temperature. Compared with the conference version of this work, this paper achieves more in-depth physical mechanism discoveries and substantial performance improvements [1]. Concurrently, to save costs and improve integration without affecting the working status of other modules, temperature sensors are often required to occupy a small area on the overall chip [2]. Especially in some mobile devices and module stack packaging, thermal management of chips has emerged as an important part of design. However, limited module footprint and miniaturized packaging constraints impose stringent requirements on temperature sensors for a small area and low power consumption [3].

Most temperature sensors’ working modules can be divided into the following two parts: temperature-sensitive circuits and analog-to-digital converters (ADCs). Based on the temperature-sensing element, the temperature-sensitive circuits can be further divided into the following three types: BJT, Complementary Metal-Oxide-Semiconductor (CMOS), and resistor-based ones [4]. BJT-based temperature sensors demonstrate superior measurement accuracy, yet their fundamental limitation resides in elevated power consumption characteristics [5,6]. Resistor-based temperature-sensitive circuits boast certain advantages in power consumption, but the overall circuit performance is critically dependent on the temperature-sensitive parameters of resistive components. Stringent fabrication precision in resistive temperature sensors (RTSs) is thus necessitated to ensure reliability and accuracy, resulting in decreased linearity and increased sensor errors [7]. Therefore, the BJT and resistor-based solutions are unsuitable for large temperature ranges, small areas, and low power consumption applications.

CMOS temperature-sensitive circuits outperform alternative implementations in terms of chip area and power efficiency due to small-sized MOS transistors and their primary operation in static states or subthreshold regions [8]. However, the nonlinear change in subthreshold region current with temperature compromises the error and resolution performance of the sensor [9]. Compared to existing temperature sensor solutions [10,11], this paper proposes a fully CMOS-structured design without large RC devices for area reduction. As for the ADC selection, the operating ambient temperature of the temperature sensor fluctuates significantly, which can degrade the conversion performance of Time-to-Digital Converter (TDC) and introduce severe nonlinearity, making it hard to guarantee stable operation [12]. Accordingly, ADC architecture with lower temperature sensitivity is adopted in this design. At the same time, the input voltage range of ADC processing has clear upper and lower limits, which is not conducive to the expansion or change in the temperature range. Therefore, frequency-domain ADC is hereby adopted for signal digitization. The circuit utilizes PFM instead of the complex ADC module used in traditional temperature sensors. In contrast to the aforementioned solutions, the PFM solution features an extensive input dynamic range. Meanwhile, it adopts a simple loop structure and delivers superior performance in chip area and power consumption [13,14].

A CMOS current mirror is utilized as temperature-sensitive circuits, and a simple PFM serves as the analog-to-digital converter [14]. This combination reduces power consumption and circuit area. However, the output of the current mirror is highly susceptible to the channel length modulation effect, and the PFM loop exhibits temperature sensitivity. It degrades the linearity and compresses the output current range of −60 °C to 40 °C, leading to a poor resolution of 500 mK.

Consequently, a cascode current mirror configuration is adopted to address the channel-length modulation effect, stabilizing output impedance through structural optimization [15]. The design further utilizes a PFM module with an improved monostable architecture, and temperature-insensitive fixed biasing networks are adopted to reduce its thermal sensitivity. By integrating these techniques, the solution maintains consistent signal chain functionality across operational temperature conditions, achieving a temperature measurement range from −60 °C to 120 °C and a power consumption of 2.9 μW. Furthermore, temperature fluctuations induce variable loop delays manifested as pulse width variations, which may lead to oscillation failure under extremely high/low temperature conditions. To reduce this influence, an improved monostable circuit is incorporated to ensure the adequate reset of each pulse and less sensitive pulse width in a wider temperature range.

However, the pulse width is exhibited as an error in the digital output. Correspondingly, this work introduces a new compensation method. This compensation adds the equivalent current of the system fixed delay and the loop thermal-related delay in the model, which more accurately eliminates these two kinds of errors from the final output, thereby reducing the error of the compensation model. The error budget consists of the following two categories of error sources: static delay errors from comparator response time and digital loop latency at normal temperatures, and dynamic thermal delays of the digital loop and delays proportional to sensor resistor drift rates. These dynamic terms can be quantified through temperature-dependent current modeling. The improved monostable circuit and correction scheme achieves a resolution of 47 mK (rms) within a further extended temperature range of −60 °C to 120 °C.

This paper is organized as follows. Section 2 introduces the circuit principle, structure, and detailed design; Section 3 describes the principle of the improved output correction; Section 4 details the implementation and measurement results of the prototype; and Section 5 provides conclusions and presents future outlooks.

## 2. Materials and Methods

### 2.1. PFM Working Principle

**Figure 1:**
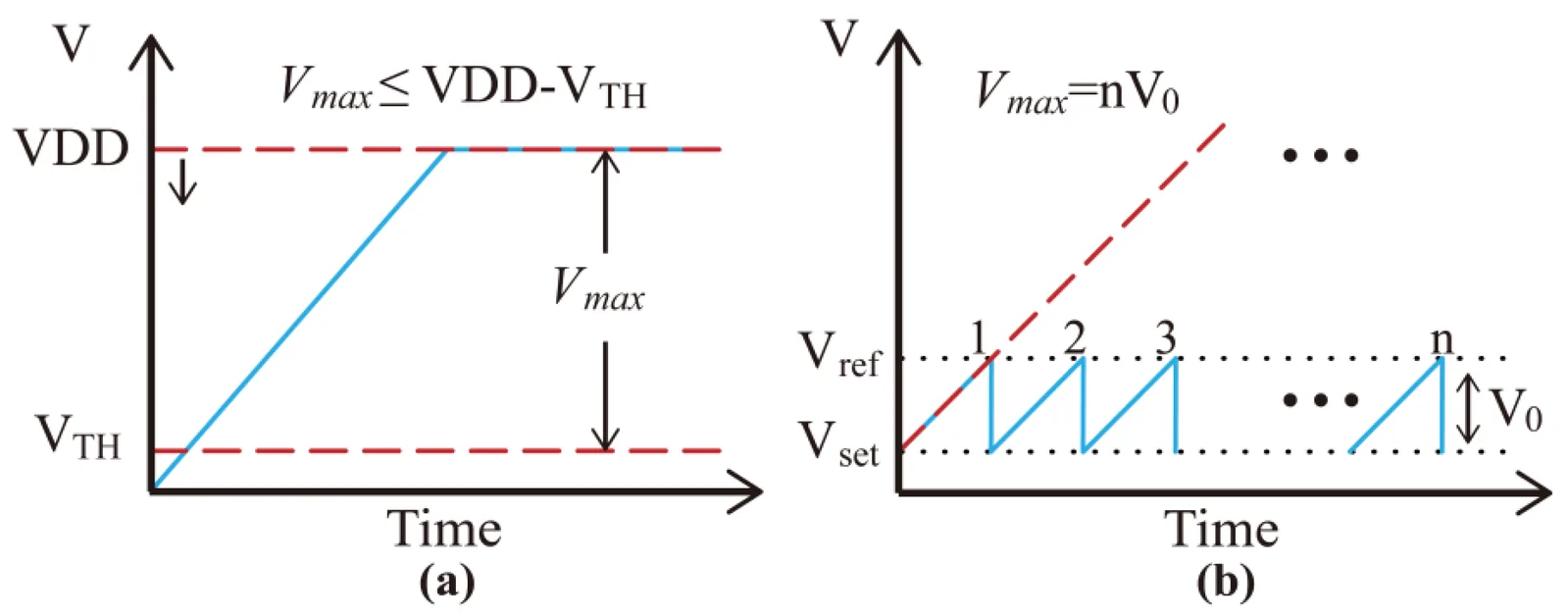
(**a**) Input voltage of traditional voltage-domain ADCs. (**b**) Input voltage of PFM.

As shown in Figure 1a, the actual maximal input swing Vmax of the traditional voltage domain ADC is affected by the supply voltage (VDD) and the noise. With the constant advancement of CMOS technology, the VDD of CMOS devices decreases continuously. The threshold voltage decreases slightly with more advanced processes, making the maximum processable voltage swing Vmax even smaller in more advanced processes, thereby reducing the temperature measurement range. As shown in Figure 1b, PFM converts voltage signals into time-domain signals via cyclic integration and reset operations combined with pulse counting, which effectively extends the equivalent processing voltage swing to n times that of the traditional swing. This allows PFM to avoid the effects of VDD and VTH on the measurable voltage range, considerably expanding the temperature measurement range [16]. Meanwhile, PFM is more inclined towards digital logic. Compared to other ADC designs, PFM suffers from severe reset noise induced by repeated KT/C noise, leading to inferior noise performance compared with other ADC architectures. However, PFM systems function reliably across wider temperature conditions while maintaining smaller circuit sizes and lower power usage. This renders them better suited for applications where stable performance in harsh environments and energy efficiency take priority over ultra-high measurement precision.

By adjusting the conversion time, PFM can obtain different resolutions for the same input. For instance, extended integration time provides higher resolution, allowing flexible tradeoffs between measurement resolution and conversion speed to accommodate diverse application demands.

### 2.2. Architecture Design

**Figure 2:**
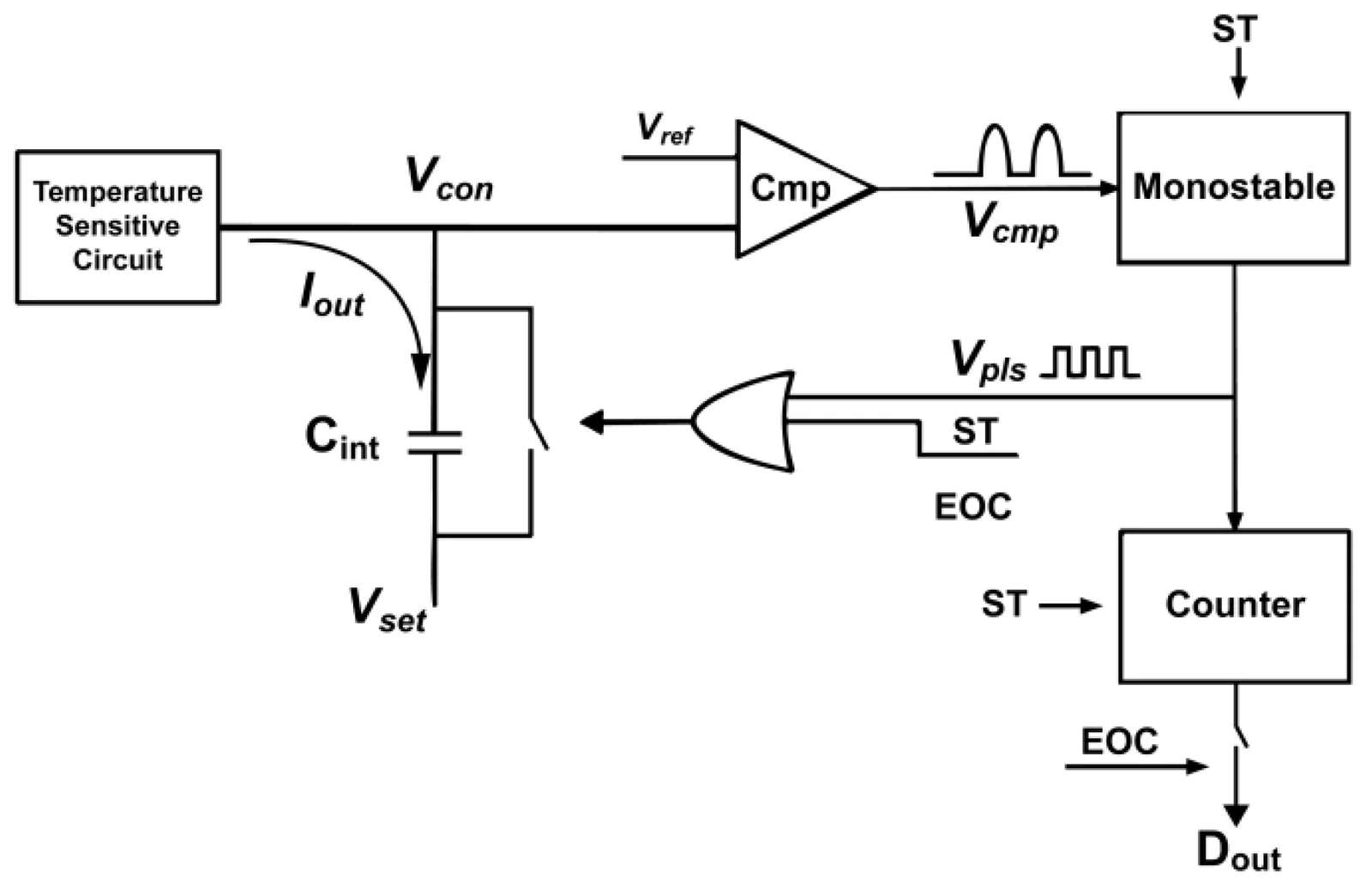
Block diagram of the proposed temperature sensor.

**Figure 3:**
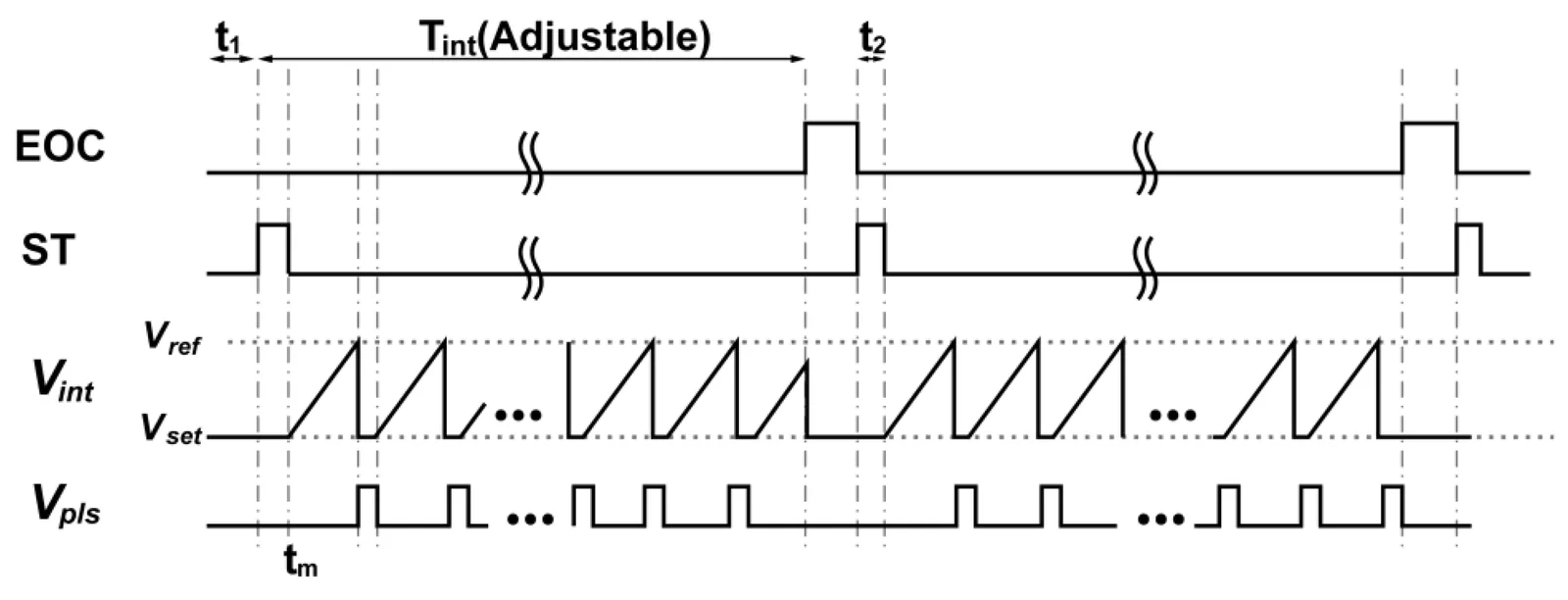
Circuit timing diagram.

Figure 2 shows the circuitry structure, including the temperature-sensitive circuit, comparator, monostable, and counter, and Figure 3 presents the timing diagram. The circuit undergoes a reset operation when ST (Start) changes from low to high, and commences operation at the falling edge of ST. The temperature-sensitive circuit generates a temperature-dependent current that flows into the integrating work capacitor, yielding an integrating voltage V_int_. When V_con_ exceeds V_ref_, the comparator flips, generating a lower-quality pulse V_cmp_ that enters the monostable module. The monostable module shapes V_cmp_ to produce a high-quality square wave V_pls_. V_pls_ enters the counter, increments the count value by 1, resets the integrating capacitor through the switch, and initiates the subsequent integration. This cycle repeats multiple times until the integration time ends. At that point, the signal EOC (End of a conversion) changes from low to high, resets the integrating capacitor, and reads out the digital value in the counter. Once EOC falls back to low, the one work frame completes and waits for the next ST signal to reset. The relationship between Dout and Iout can be expressed as follows:

(1) $$D_{out}=\frac{I_{out}T_{con}}{C_{int}(V_{ref}\;–{\;V}_{set})}$$

Considering that the output node voltage of the temperature-sensitive circuit is V_int_, its magnitude must be constrained to prevent the current mirror from operating in its nonlinear region. Accordingly, the selection of V_set_ and V_ref_ should be close to the threshold voltage. In this case, a two-stage, five-transistor amplifier with PMOS input transistors is adopted to achieve the best bandwidth and gain.

In the feedback loop, the presence of comparator propagation delay and the use of resistors without zero temperature coefficient will induce thermal drift-induced resistance variations under different temperatures, leading to certain non-ideal characteristics in the system. These non-ideal effects will compromise the system’s linearity performance. A detailed analysis will be presented in Section 3.

### 2.3. Temperature Sensor

The basic principle of converting temperature into current information stems from the fact that the current flowing through a CMOS transistor operating in the sub-threshold region exhibits a proportional relationship with temperature, as expressed by the formula below:

(2) $$I=\mu Cox(\frac{W}{L})V_{T}^{2}EXP(\frac{V_{GS}}{nV_{T}})$$

(3) $$V_{T}=\frac{kT}{q}$$

where μ is carrier mobility, C_ox_ is the gate oxide capacitance of the CMOS transistor, W/L is the length-to-width ratio of the CMOS transistor, V_GS_ is the gate-source voltage of the CMOS transistor, k is the Boltzmann constant, T is the absolute temperature, and q is the charge of one electron.

**Figure 4:**
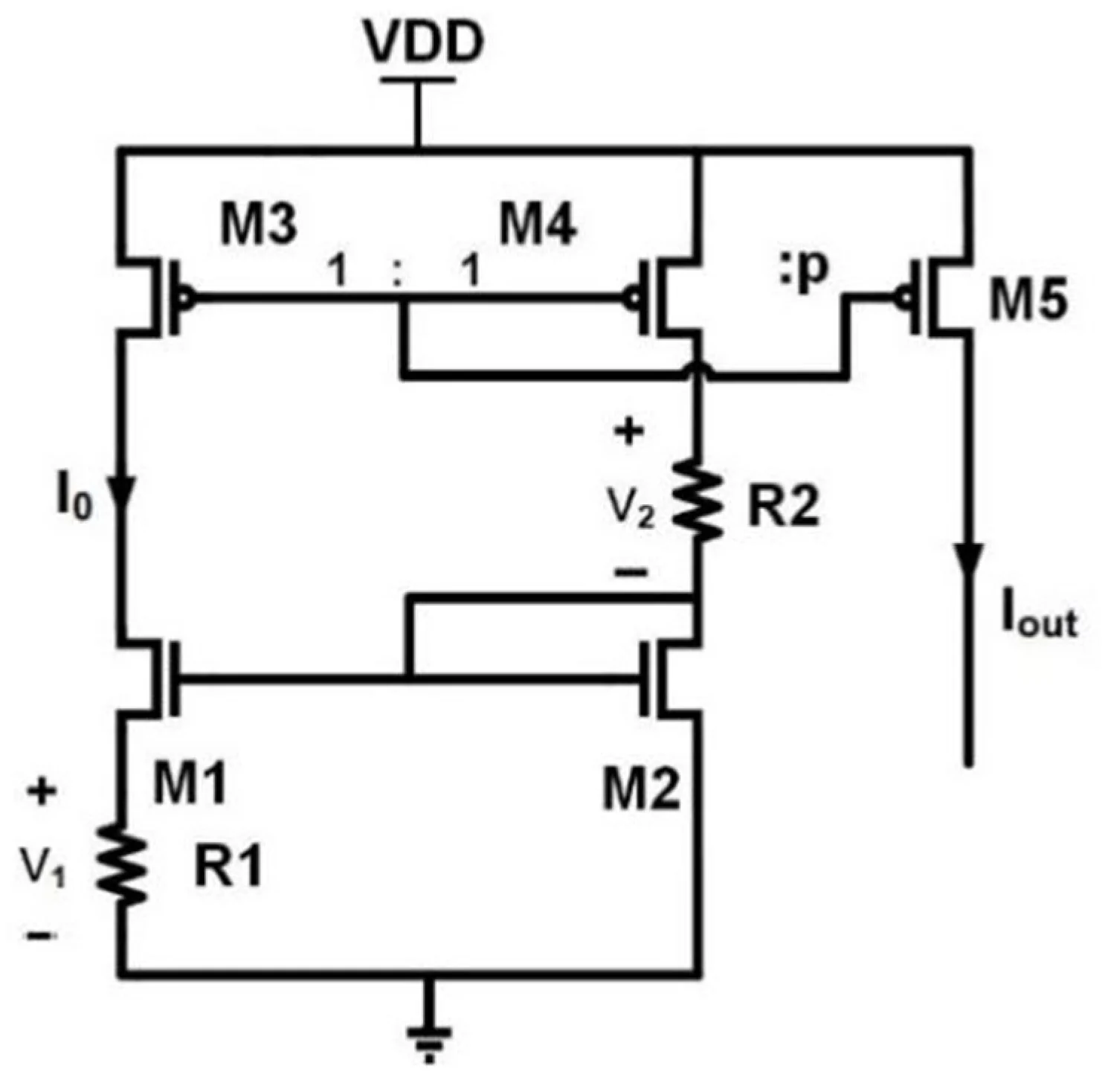
Schematic of the simple cascode current mirror.

**Figure 5:**
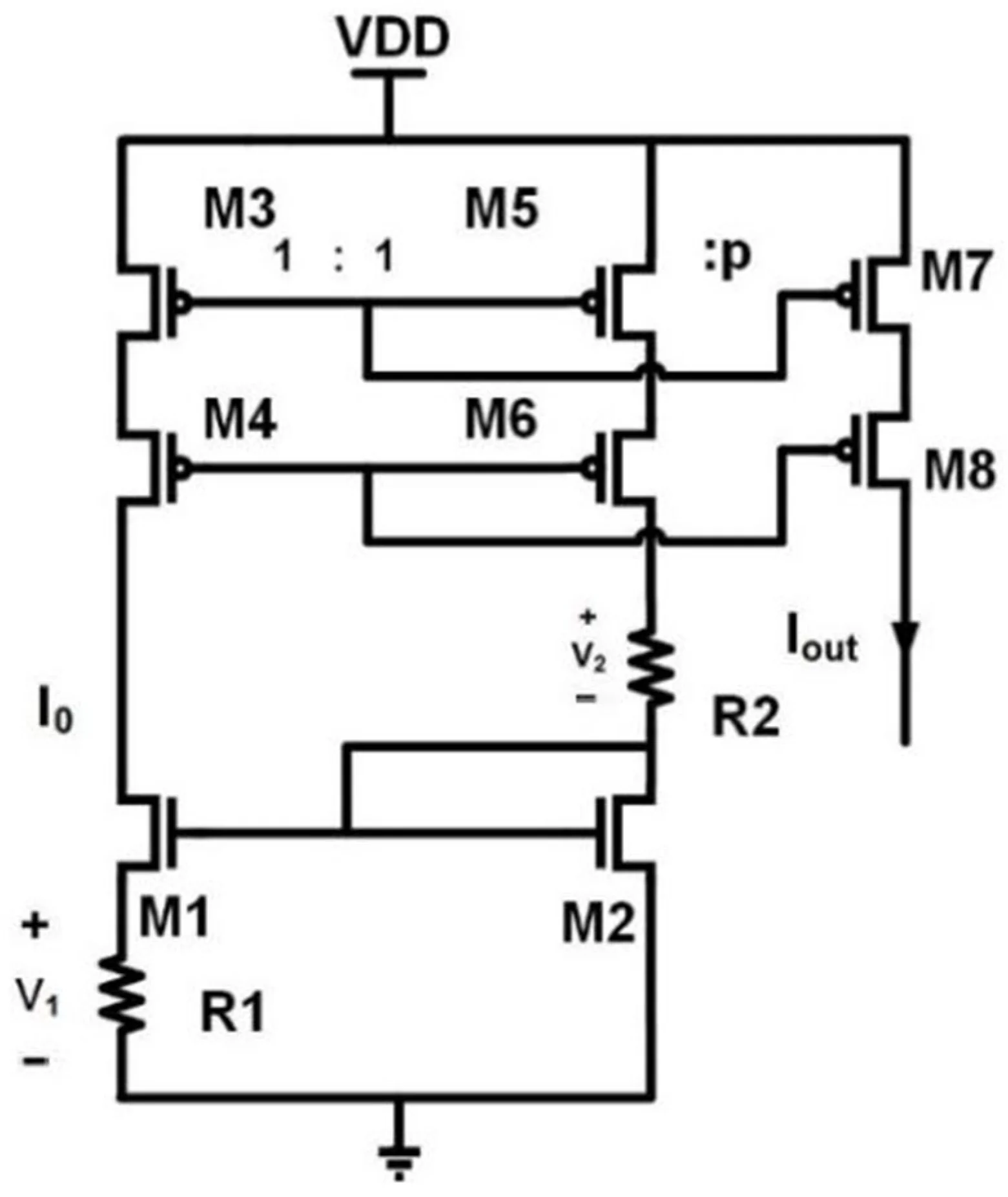
Schematic of the proposed improved cascode current mirror.

**Figure 6:**
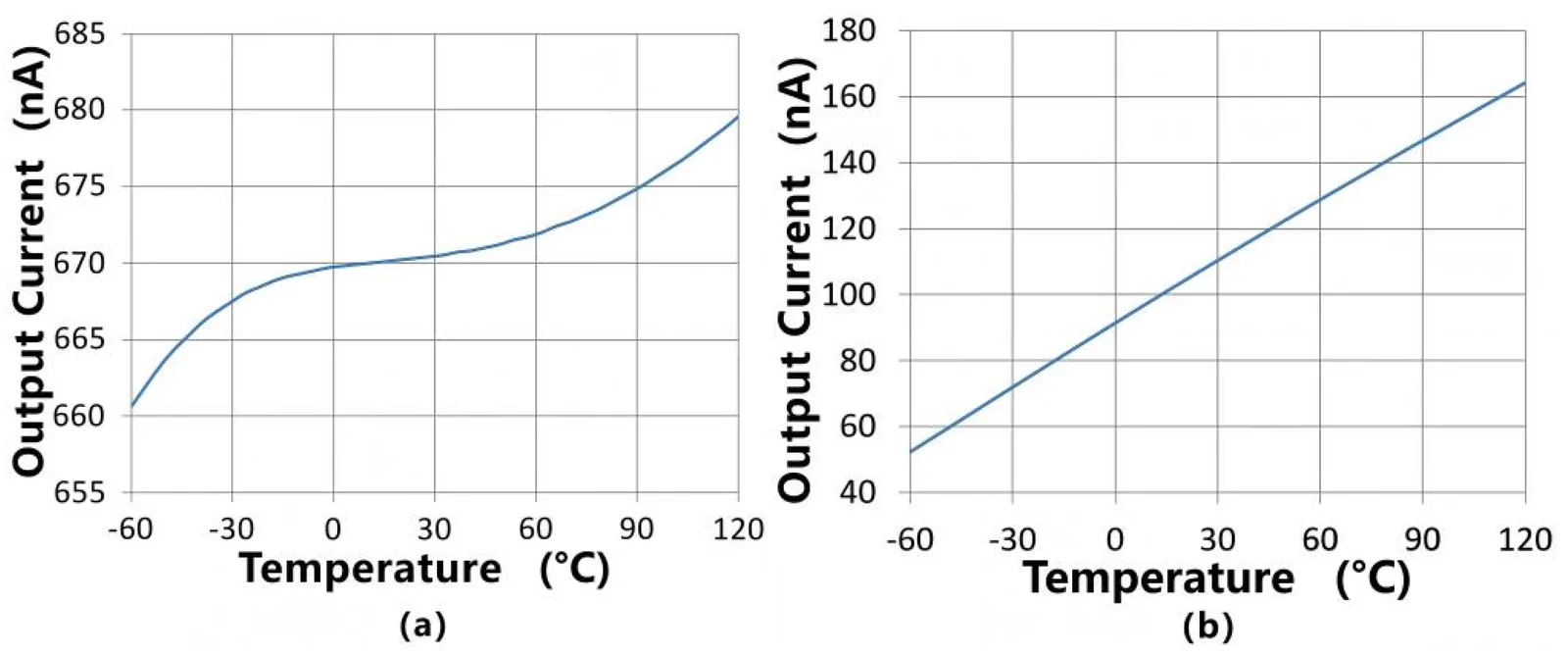
(**a**) Current output of the simple cascode current mirror. (**b**) Current output of the improved cascode current mirror.

A current mirror is shown in Figure 4. Experimental results indicate that a simple current mirror suffers from channel-length modulation induced by periodic fluctuations in Vint. This narrows the output range of the current source, exacerbates channel-length modulation errors, and decreases output linearity. Under these conditions, Figure 5 shows the temperature-sensitive current generation with an improved self-biased cascade current mirror used in this work. The output current is copied through a simple mirroring relationship without protection, as presented in Figure 6a. The output of improved cascode current mirror is shown in Figure 6b. Using the cascade current mirror improves the linearity and swing of the current source output. A pair of MOS transistors and resistors generate the temperature-sensitive current. Due to the current mirror, the current of M_1_ is equal to that of M_2_. The current flowing through the resistor R_1_ exhibits a linear temperature dependence. This is attributed to the temperature dependence of the MOS transistor characteristic voltage V_T_. In temperature-sensitive circuitry, the predominant error mechanisms stem from the following two thermal dependencies: resistive variations proportional to ambient temperature and threshold voltage shifts in transistors induced by the coupling of temperature and body effects.

The resistors used in the design exhibit a specific temperature coefficient, and the functional relationship between the resistance and temperature can be expressed as follows [14]:

(4) $$R_{1}(T)=R_{1}[1+K_{R}(T-T_{0})]$$

where KR is the thermal sensitivity coefficient.

Moreover, temperature-induced shifts in the threshold voltage of CMOS transistors introduce nonlinearity to the change in temperature-sensitive current. The relationship between the threshold voltage and temperature can be expressed as follows:

(5) $$V_{TH}(T)=V_{TH}(T_{0})+K_{1}(\frac{T}{T_{0}}-1)+K_{2}V_{BS}(\frac{T}{T_{0}}-1)$$

where K1 and K2 are process parameters, and VBS is the voltage between the substrate and the source terminal of M1 and M2 (assuming that two transistors are manufactured under the same process). Under this condition, the final temperature-sensitive current can be expressed as follows:

(6) $$I_{\mathrm{OUT}}\;=\;\frac{\mathrm{pkT}}{\mathrm{qR}[1\;+\;(\frac{K_{2}}{T_{0}}\;+\;K_{R})(T-T_{0})]}$$

where p denotes the current mirror copy ratio.

Accordingly, no direct linear relationship exists between T and I, which requires linear correction to obtain a linear function output with temperature. Considering the complexity of the calibration model, the off chip multi-point calibration method is hereby employed, with the specific content introduced in Section 3.

### 2.4. Monostable Circuits

**Figure 7:**
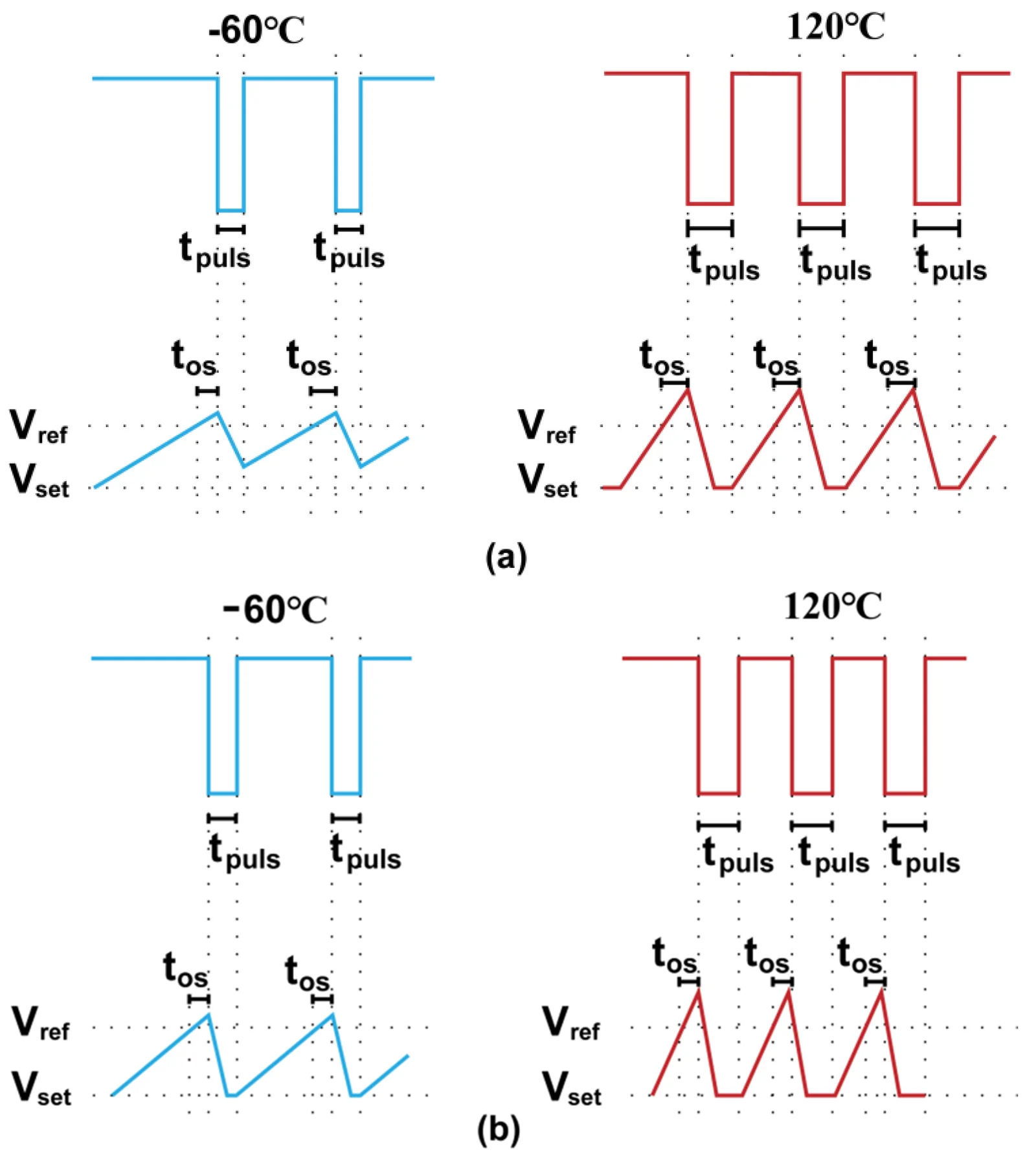
(**a**) Workflow without monostable module. (**b**) Workflow with monostable module.

The data transmission loop must maintain reliable operation across varying thermal conditions in temperature sensor circuits. Conventional delay-line-based architectures suffer from significant temperature-dependent limitations in generating reset pulses for integration capacitors. As shown in Figure 7a, at lower temperatures, the reduced propagation delay of logic gates results in excessively narrow reset pulses, which may fail to fully discharge the integration capacitor, leading to residual voltage accumulation and subsequent measurement inaccuracies. Conversely, at elevated temperatures, increased circuit delay widens the pulse width beyond its optimal operating range. This results in extended reset phases, which exacerbate timing mismatches and elevate nonlinear errors.

Integrating a monostable model into the data transmission link holds considerable significance in addressing these challenges. As shown in Figure 7b, the monostable module achieves temperature-stable reset pulses primarily through an externally stabilized current bias, which maintains precise charge/discharge rates in its timing circuitry. By replacing traditional delay-dependent logic chains with a current-controlled timing mechanism, this design decouples pulse width from thermal fluctuations. A stabilized bias current ensures consistent capacitor-charging characteristics across temperature extremes. Such stability guarantees complete capacitor resetting at low temperatures and avoids over-extension at high temperatures, effectively minimizing timing-induced errors.

**Figure 8:**
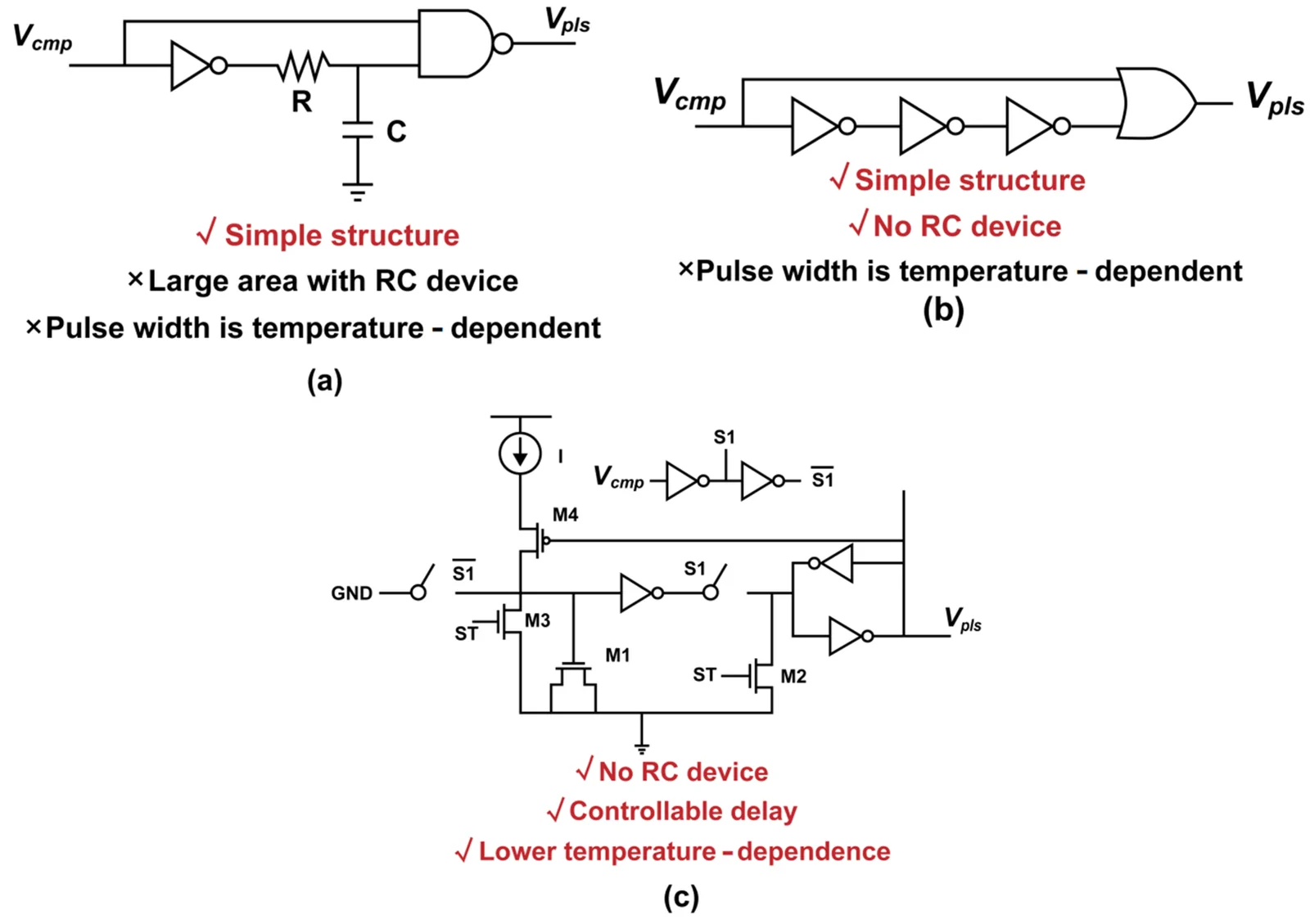
(**a**) Traditional monostable circuit. (**b**) Monostable circuit in [14]. (**c**) Improved monostable circuit.

Figure 8a shows the traditional monostable circuits with RC device. RC components exhibit strong temperature-dependent delay characteristics. Therefore, the quality of the output pulse may be affected by changes in temperature, resulting in changes in pulse width and amplitude. Accordingly, the reset pulse generated by the RC delay module may fail to fully discharge the integration node, resulting in lock-up and loss of function. The inverter-based delay chain monostable module in [14] remains sensitive to temperature. It is prone to nonlinear delay accumulation and incomplete reset, which restricts its temperature range scalability and circuit stability. As shown in Figure 8c, when ST arrives, the module output is pulled down, the switch connecting the constant current source to the MOS capacitor is turned on, and the gate voltage of M_1_ rises accordingly. When the M_1_ gate voltage reaches the inverter flip-flop threshold, the existing switch is closed, and the output signal is pulled up. The module generates square-wave pulses with adjustable pulse width to reset the integral capacitor. The improved monostable circuit isolates the input and output signals, removing temperature-dependent interference from RC devices. It also effectively suppresses the effects of glitches and noise in the input signal on the output signal, ensuring high integrity of the reset signal. This circuit completely resets the voltage on the built-in integration capacitor at the beginning of each frame and the arrival of each pulse. This operation effectively avoids charge accumulation on the integration capacitor, resulting in errors or latches, thus ensuring continuous, reliable signal transmission. Meanwhile, it prevents the impact of glitches in the V_cmp_ on the V_pls_.

The reset signal integrity of three signal chains (delay line, simple monostable, and improved monostable under varying temperatures) reveals critical thermal dependencies. At −60 °C, the basic monostable fails to operate properly due to compressed reset pulse widths. In delay chains, a short reset pulse prevents proper integration capacitor resetting. At 30 °C, all three signal chains exhibit nominal functionality, stable reset characteristics, and minimal timing deviations. However, at 120 °C, excessive delays from both simple monostable and delay lines exacerbate nonlinearity and errors. The improved monostable module delivers superior temperature resilience. It sustains consistent reset quality across extremes and suppresses nonlinear distortions.

## 3. Results

### 3.1. Output Correction

The preceding section has analyzed the change in MOS transistor Vth, accounting for variations in temperature monitoring and the output current offset caused by deviations in resistance with temperature in the temperature measurement circuit. When only considering the non-temperature-related delay present in the circuit, the relationship between Dout and T can be compensated and corrected using the following formula:

(7) $$T\;=\;\frac{\left(D_{\mathrm{OUT}}-B)(1-K_{\mathrm{TC}}T_{\mathrm{con}}\right)}{A-K_{\mathrm{TC}}(D_{\mathrm{OUT}}-B)}$$

(8) $$A=\frac{\mathrm{pk}T_{\mathrm{con}}}{qR_{1}C_{\mathrm{int}}(V_{\mathrm{con}}{–\;V}_{\mathrm{set}})}$$

(9) $$B=-\frac{T_{\mathrm{con}}I_{\mathrm{os}}}{C_{\mathrm{int}}(V_{\mathrm{con}}-V_{\mathrm{set}})}$$

where I_os_ represents the current form of non-temperature-related delay in the system.

**Figure 9:**
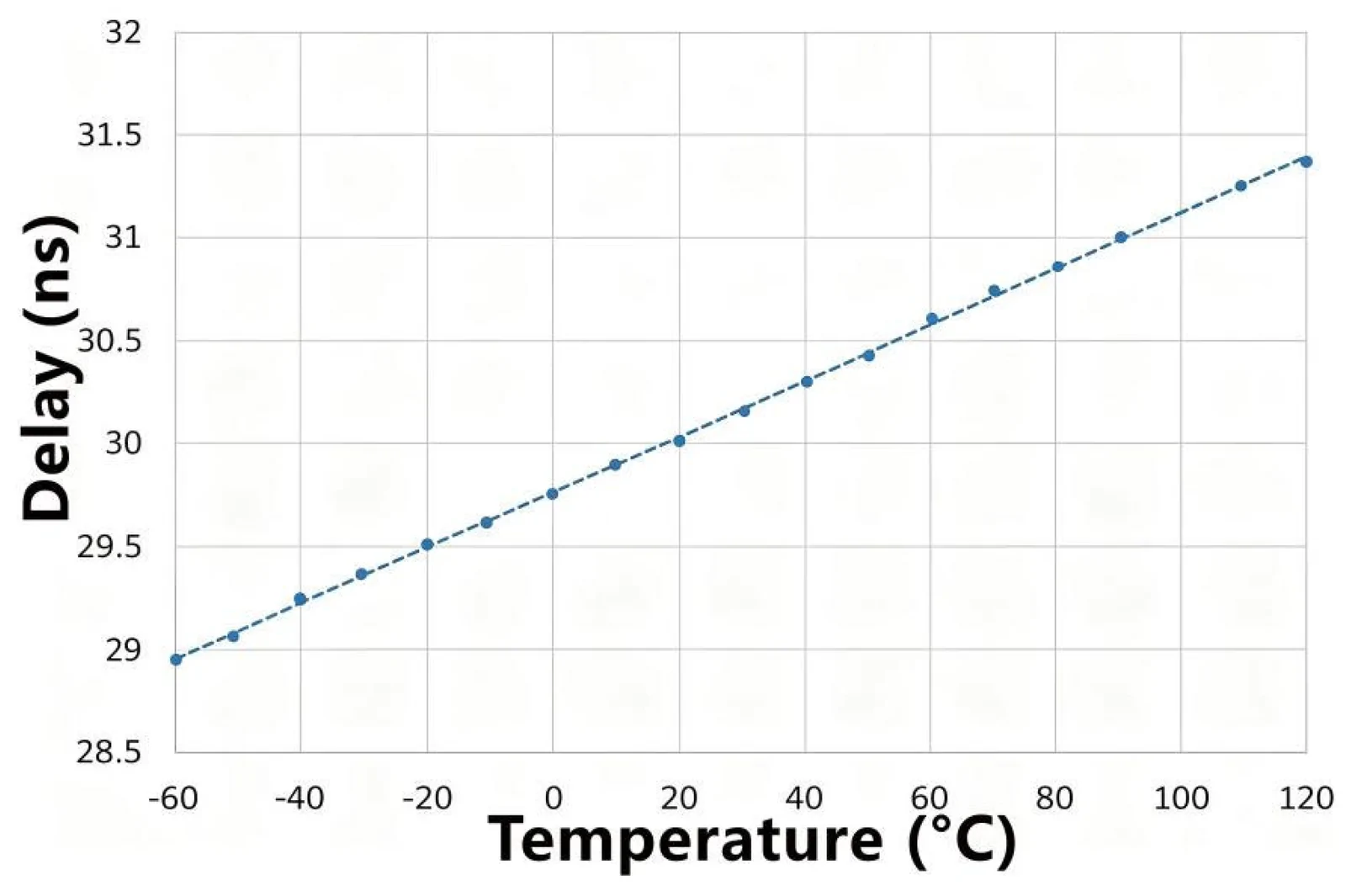
System delay at different temperatures.

However, in the above situation, it is challenging to directly quantify the comparator and circuit transmission delay. These delays vary with changes in the operating temperature of the circuit, generating a temperature-depending delay variable. In the circuit simulation, the delay caused by the comparator and transmission link varies with temperature, as shown in Figure 9. This delay is treated as a constant in the previous calibration model, and the error caused by this delay is amplified with expanding temperature range, resulting in an increase in sensor error.

To compensate for the temperature-related delay, the time delay tos independent of temperature and the thermoelectric time delay t_temp_ is introduced as the temperature-related delay compensation, and its magnitude is proportional to T:

(10) $$t_{\mathrm{temp}}\;=\;\gamma T$$

where γ is the temperature correlation coefficient, and its magnitude should be determined through calibration. Concurrently, its current flows in the opposite direction to the integrated current. In this case, the expression for a single integration period t0 can be expressed as follows:

(11) $$t_{0}\;=\;\frac{C_{\mathrm{int}}(V_{\mathrm{con}}-V_{\mathrm{set}})}{I_{0}}+t_{os}+t_{temp}$$

By substituting Equations (10) and (11) into Equation (1), a new compensation correction formula can be obtained as follows:

(12) $$T\;=\;\sqrt{b\;+\;\frac{\alpha^{2}}{4}}\;+\;\frac{a}{2}$$

where

(13) $$\alpha =(\frac{K_{tc}(V_{\mathrm{con}}-{\;V}_{\mathrm{set}})BD_{\mathrm{out}}-At_{OS}D_{\mathrm{out}}-AT_{\mathrm{con}}}{\gamma AD_{\mathrm{out}}})$$

(14) $$b=\frac{K_{tc}(V_{\mathrm{con}}-V_{\mathrm{set}})(D_{\mathrm{out}}-BD_{\mathrm{out}}T_{0})}{\gamma AD_{\mathrm{out}}}$$

By measuring Dout at four different temperature points, (T1, Dout1), (T2, Dout2), (T3, Dout3), (T4, Dout4) can be gained. By substituting the temperature Tx and digital readout Doutx of the four points into Equation (12), accurate correction can be performed for parameters a, b, γ, and KTC, which remarkably reduces system errors. Given that the nonlinear calibration introduced by the model relies on multi-point calibration, the required arithmetic module to complete on chip is relatively large. Consequently, off-chip calibration is utilized.

### 3.2. Measurement Results

**Figure 10:**
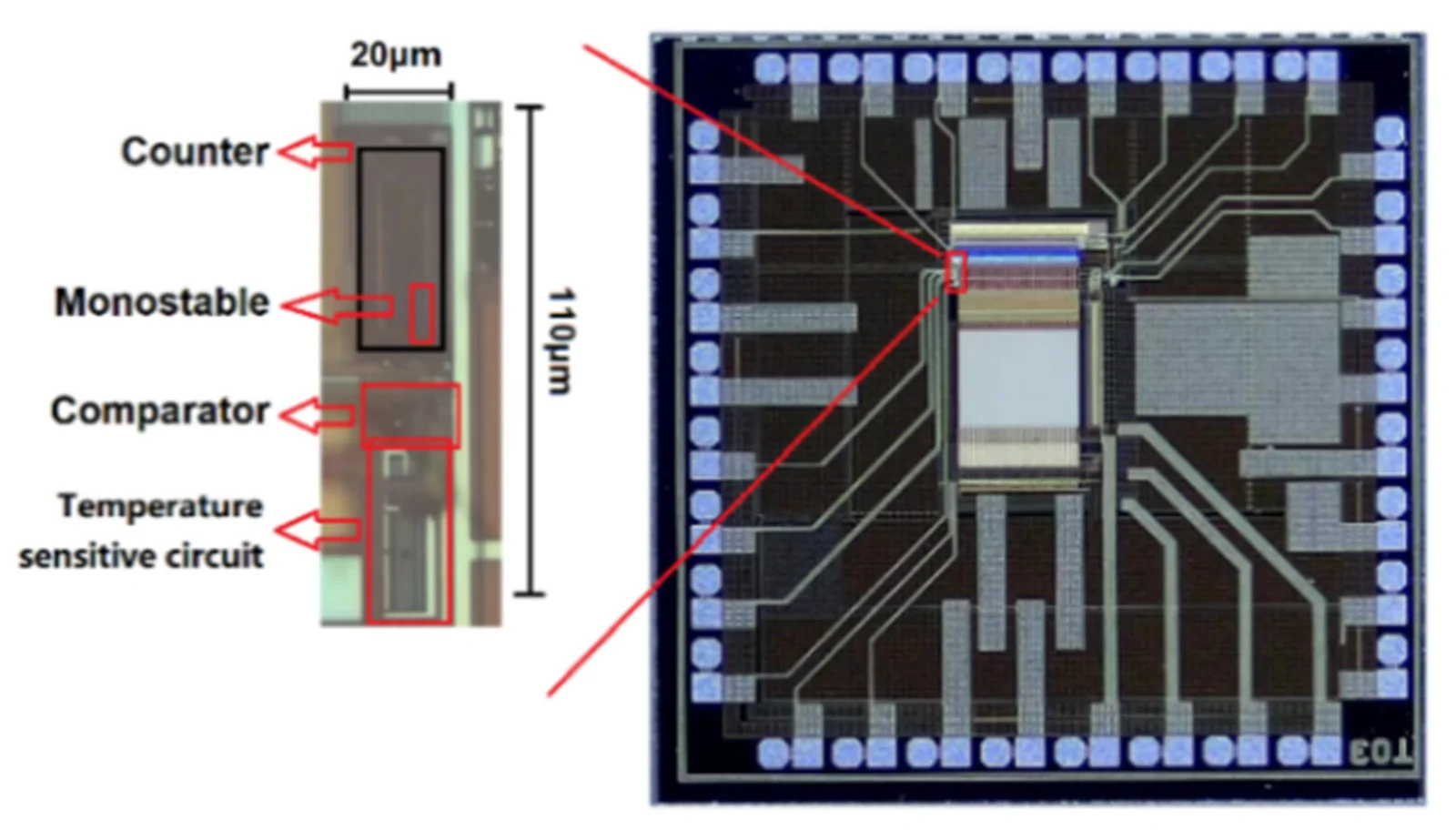
Die photograph.

The proposed circuits are implemented in the 180 nm CMOS process, and a chip micrograph integrating the presented sensor alongside other circuits is shown in Figure 10. The temperature sensor occupies a core area of 2200 μm^2^.

**Figure 11:**
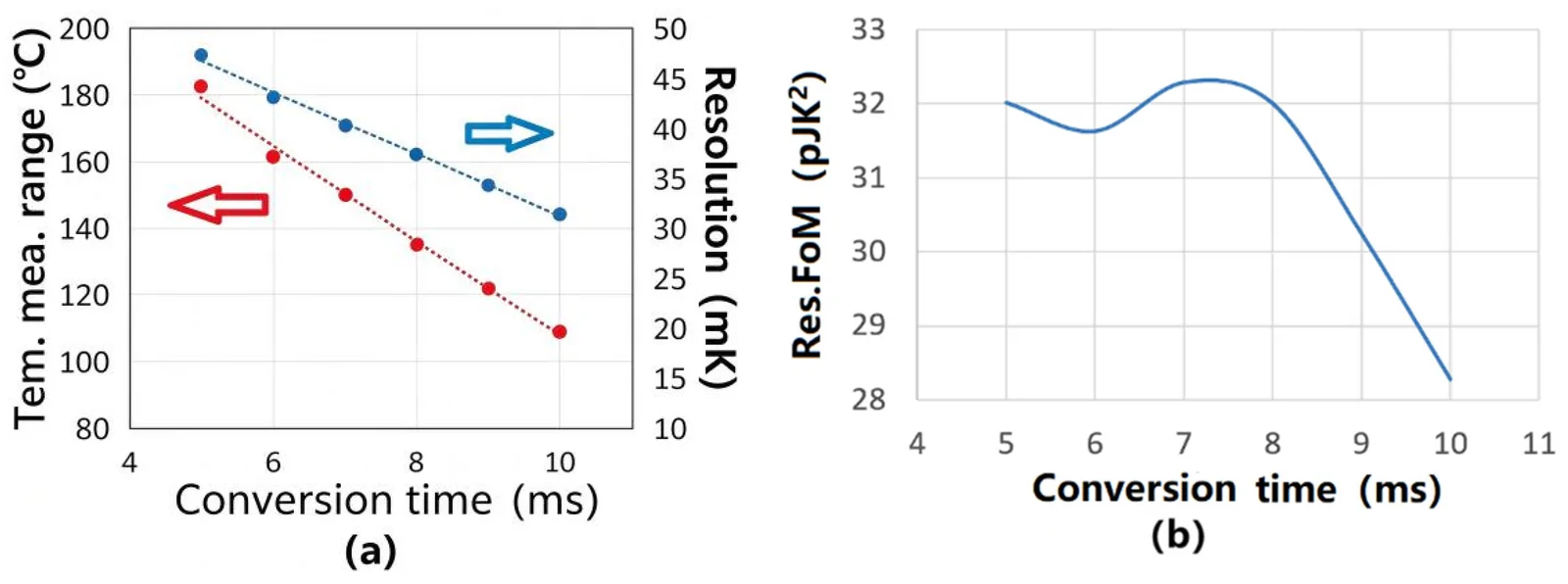
(**a**) The resolution and temperature measurement range varies with conversion time. (**b**) The change in Res. FoM with integration time.

To evaluate the performance, eight samples are measured. By changing the conversion time of the sensor, the sensor achieves different temperature measurement ranges, yet at the cost of resolution. As shown in Figure 11a, when the conversion time changes from 5 ms to 10 ms, the temperature measurement range of the sensor narrows from 180 °C to approximately 110 °C, while the resolution improves from 47 mK to 31 mK. The maximum measurement temperature is limited by the temperature chamber, whose highest temperature is merely 120 °C. With advanced testing hardware, the sensor can achieve a broader temperature measurement range.

However, the temperature chamber exhibits constrained performance, featuring temperature fluctuations of about ±0.5 °C and an error of ±2 °C. To solve this limitation, a calibrated Pt thermocouple temperature sensor (with an error of 0.3 °C) is adopted to accurately calibrate the temperature sensor. In the experiment, the platinum electrode of the thermocouple temperature sensor is placed in the box of the temperature sensor and tightly adheres to the temperature sensor, enabling the thermocouple to accurately measure the working temperature of the sensor chip.

The resolution FoM with different conversion times is shown in Figure 11b. Although the trade-off between resolution and temperature measurement range should be considered, the design retains favorable FoM performance. The peak FoM degradation occurs at a conversion time of 5 ms.

**Figure 12:**
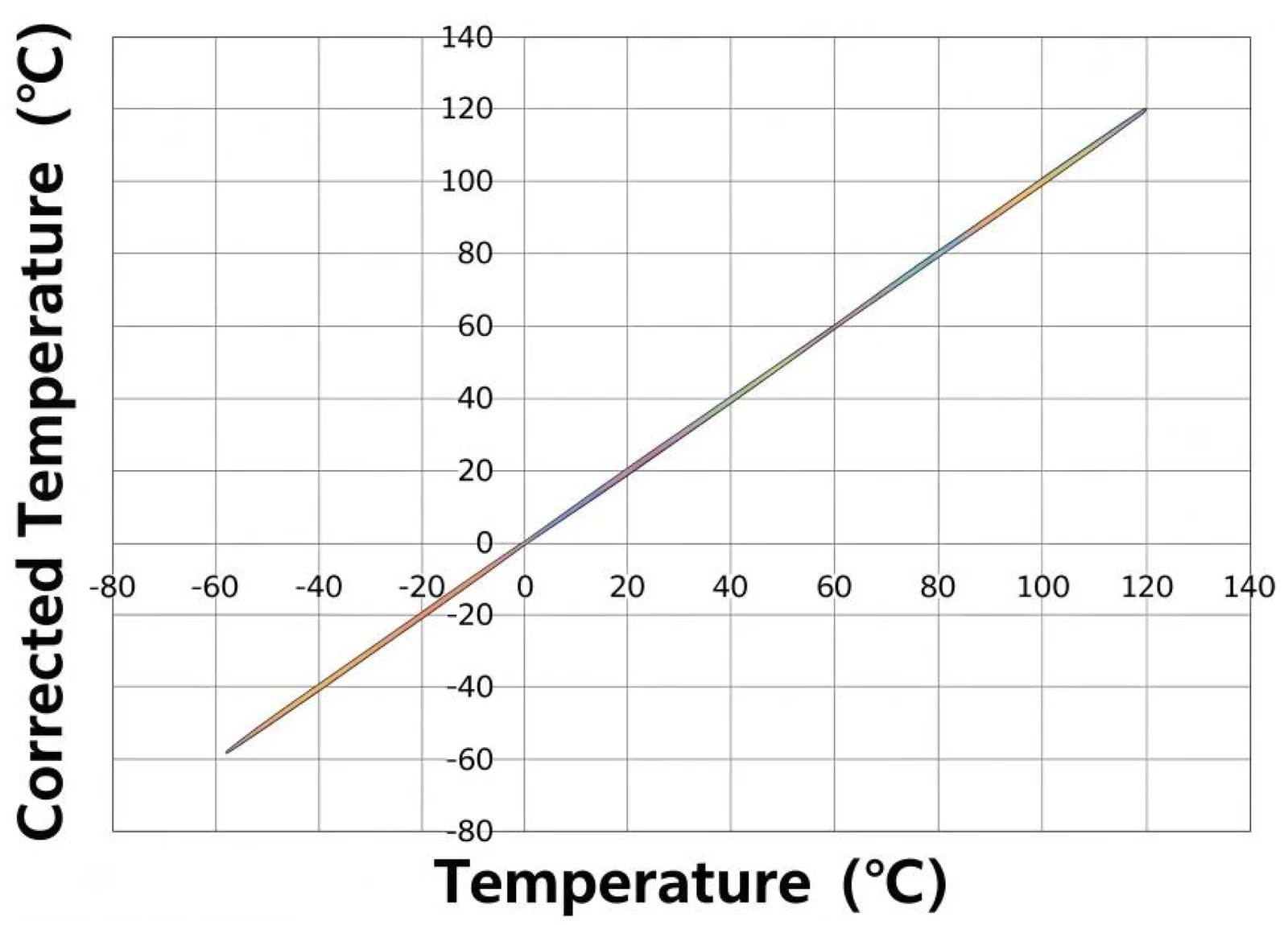
Corrected output values of 8 samples (Tcon = 5 ms).

**Figure 13:**
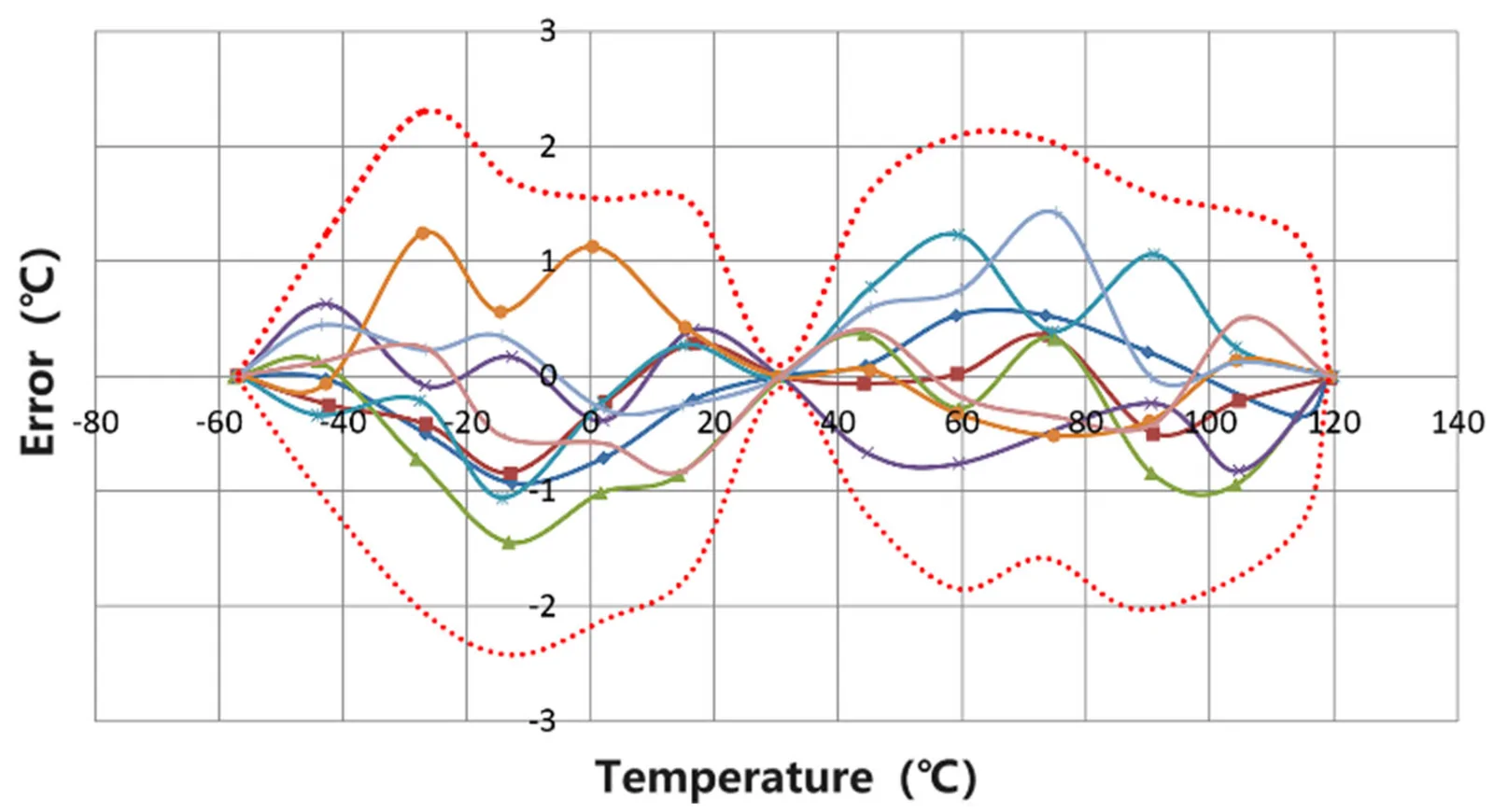
Circuit error with the original calibration model (including 3σ errors under Tcon = 5 ms, each solid line represents one sample, with a total of eight samples).

**Figure 14:**
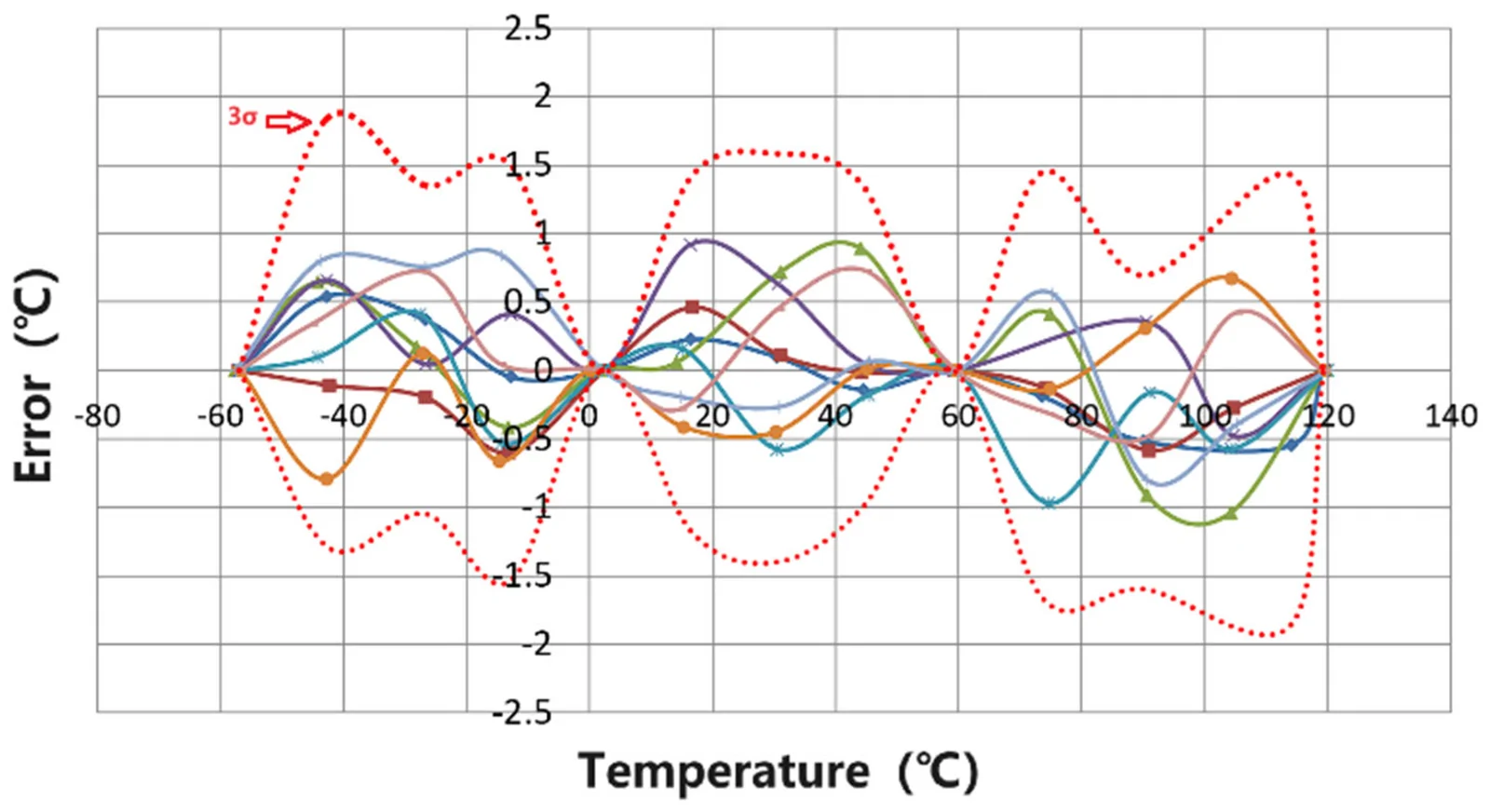
Circuit error with the improved calibration model (including 3σ errors under Tcon = 5 ms, each solid line represents one sample, with a total of eight samples).

For a conversion time of 5 ms, eight samples are measured from −60 °C to 120 °C, with a step of 15 °C. Due to the correctable nonlinear factors described in Section 3, the final digital output of the circuit is not linear to the temperature. The non-linearity correction is performed according to (12), and the output is shown in Figure 12. The measured resolution (RMS) is 47 mK. Upon correction, the output achieves a favorable fitting with the actual temperature. As shown in Figure 13, the previous model corrected by Equation (7), the circuit shows a temperature error of +2.4/−2.3 °C over the range of −60 °C to 120 °C. The improved model calibrated using (12) exhibits a corrected error, as shown in Figure 14. This correction delivers an error of −1.9/+1.8 °C. The refined model achieves roughly 20% reduction in the overall system error.

**Figure 15:**
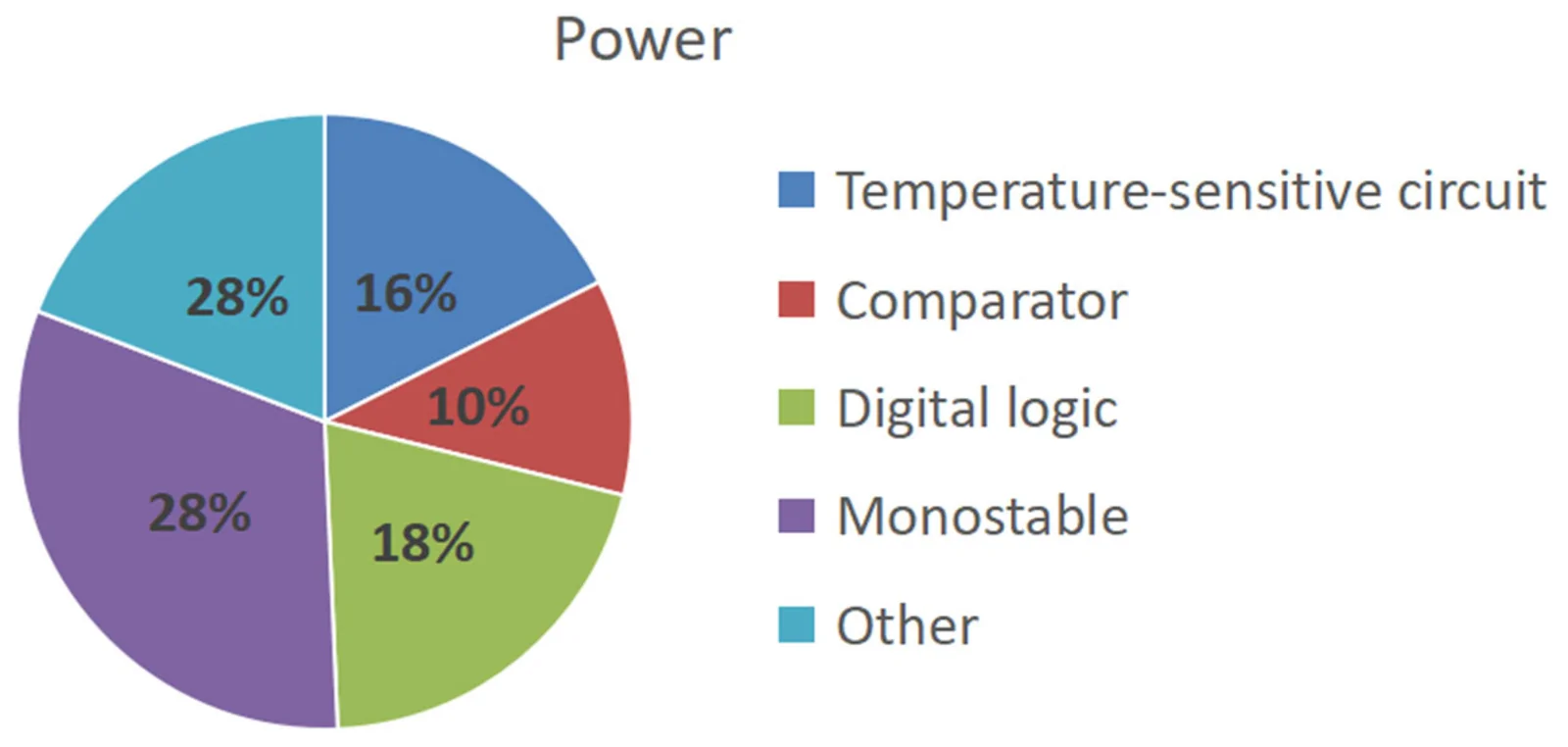
Power consumption breakdown of the presented temperature sensor chip.

The circuit operates under a 1.8 V analog voltage and a 1.2 V digital voltage supply, with a total power consumption of 2.86 μW. The power consumption ratio of each module in the circuit is shown in Figure 15. As the digital circuits dissipate roughly half of the power, the proposed sensor can consume less power in an advanced process. The comparison between this work and other similar studies is summarized in Table 1.

## 4. Discussion

The proposed PFM-based temperature sensor achieves a wide temperature range of −60 °C to 120 °C, alongside ultra-low power and small area. The improved cascode current mirror and monostable circuit effectively suppress nonlinearity and temperature-induced delay errors. The off-chip multi-point calibration further reduces the measurement inaccuracy to ±1.9 °C. Its quasi-digital time-domain architecture enables seamless scaling to advanced CMOS technologies, yielding superior power and area performance in smaller processes. The achieved FoM values demonstrate competitiveness among state-of-the-art low-power wide-range temperature sensors.

Additionally, this temperature sensor exhibits strong thermal robustness across extreme temperature environments ranging from −60 °C to 120 °C, which is mainly guaranteed by two optimized circuit modules. The improved cascode current mirror suppresses channel length modulation effects, delivering stable temperature-sensitive current output without obvious shrinkage of current swing over the full temperature range. Meanwhile, the redesigned monostable circuit eliminates temperature-dependent pulse width drift, avoiding incomplete reset and oscillation failure under cryogenic or high-temperature conditions.

Measured results further verify its reliability as follows: eight chip samples show consistent performance after multi-point off-chip calibration, with the maximum overall measurement error controlled within +1.8 °C/−1.9 °C (3σ). Even with a short 5 ms conversion time for low-latency detection, the sensor maintains a fine resolution of 47 mK without functional failure. Benefiting from the RC-free quasi-digital PFM architecture, the circuit avoids severe thermal drift caused by passive components, making it robust for aerospace and cryogenic detection scenarios with drastic temperature fluctuations.

The sensor possesses unique compatibility with emerging flexible gas sensing platforms. It can be monolithically co-integrated with flexible WO_3_/MoS_2_ heterojunction NO_2_ gas sensing readout circuits to deliver real-time on-chip thermal compensation. Such heterojunction NO_2_ detectors suffer severe temperature-induced response drift from −60 °C to 120 °C [22], which drastically undermines gas detection precision. Benefiting from the ultra-small footprint and microwatt-level power draw, this temperature sensor imposes minimal power-area overhead on wearable, aerospace and industrial low-power multi-gas intelligent sensing nodes.

## 5. Conclusions

This paper implements a wide-range PFM-based CMOS temperature sensor using a 180 nm process. The improved cascode current mirror and redesigned monostable circuit suppress channel-length modulation and temperature-dependent reset pulse drift, enhancing linearity and stability across the full temperature range. The sensor features a 2200 μm^2^ active area and 2.9 μW power consumption. At a 5 ms conversion time over −60 °C to 120 °C, it reaches 47 mK resolution, state-of-the-art resolution and area FoM values and restricts calibrated 3σ error within ±1.9 °C. Conversion time can be adjusted to balance measurement range and resolution, and its quasi-digital structure allows straightforward porting to advanced process nodes.

## Figures and Tables

**Table 1:** Benchmark comparison with prior art temperature sensors.

	[14]	[17]	[18]	[19]	[20]	[21]	This Work
Type	CMOS	CMOS	CMOS	BJT	BJT	BJT	CMOS
Technology (nm)	130	180	180	180	180	130	180
Range (°C)	−60~40	−10~100	−20~80	−55~125	−50~110	−40~125	−60~120
Inaccuracy (°C)	2	4.3	2.1	0.3	3.1	1.1	+1.8/−1.9
Area (μm^2^)	1400	120,000	74,000	250,000	6000	86,000	2200
Resolution (mK)	500	300	94	1.8	92	60	47
Conversion time (ms)	1000	200	839	128	0.08	1.3	5
Power (μW)	0.15	0.0005	0.011	0.8	61.2	39.7	2.86
Res. FoM (pJK^2^) *	37,500	9	190	0.3	41	185	32
Area. FoM/(pJK^2^mm^2^) **	52.50	1.08	14.06	0.08	0.25	15.91	0.06

* Res. FoM = Power × Conversion time × (Res)^2^. ** Area. FoM = Power × Conversion time × (Res)^2^ × Area.

## Data Availability

The data generated in this study include sensor-output data and data produced during the analysis process. The relevant data are available from the corresponding author (the first author) upon reasonable request.
